# Expression and mutation analysis of the discoidin domain receptors 1 and 2 in non-small cell lung carcinoma

**DOI:** 10.1038/sj.bjc.6603614

**Published:** 2007-02-13

**Authors:** C E Ford, S K Lau, C Q Zhu, T Andersson, M S Tsao, W F Vogel

**Affiliations:** 1 Department of Laboratory Medicine and Pathobiology, Faculty of Medicine, University of Toronto, Toronto, Ontario, Canada; 2 Division of Experimental Pathology, Department of Laboratory Medicine, Lund University, University Hospital Malmö, Malmö, Sweden; 3 Department of Medical Biophysics, University of Toronto, Toronto, Ontario, Canada; 4 University Health Network, Ontario Cancer Institute and Princess Margaret Hospital, Toronto, Ontario, Canada

**Keywords:** lung cancer, DDR1, DDR2, mutation

## Abstract

The discoidin domain receptors, (DDR)1 and DDR2, have been linked to numerous human cancers. We sought to determine expression levels of DDRs in human lung cancer, investigate prognostic determinates, and determine the prevalence of recently reported mutations in these receptor tyrosine kinases. Tumour samples from 146 non-small cell lung carcinoma (NSCLC) patients were analysed for relative expression of DDR1 and DDR2 using quantitative real-time PCR (qRT-PCR). An additional 23 matched tumour and normal tissues were tested for differential expression of DDR1 and DDR2, and previously reported somatic mutations. Discoidin domain receptor 1 was found to be significantly upregulated by 2.15-fold (*P*=0.0005) and DDR2 significantly downregulated to an equivalent extent (*P*=0.0001) in tumour *vs* normal lung tissue. Discoidin domain receptor 2 expression was not predictive for patient survival; however, DDR1 expression was significantly associated with overall (hazard ratio (HR) 0.43, 95% CI=0.22–0.83, *P*=0.014) and disease-free survival (HR=0.56, 95% CI=0.33–0.94, *P*=0.029). Multivariate analysis revealed DDR1 is an independent favourable predictor for prognosis independent of tumour differentiation, stage, histology, and patient age. However, contrary to previous work, we did not observe DDR mutations. We conclude that whereas altered expression of DDRs may contribute to malignant progression of NSCLC, it is unlikely that this results from mutations in the DDR1 and DDR2 genes that we investigated.

Lung cancer is the leading cause of cancer-associated deaths worldwide and has one of the poorest prognoses among all cancer types. Non-small cell lung carcinoma (NSCLC) comprises approximately 80% of lung cancer, and its overall 5-year survival rate is 15%. Early stage tumours are treated primarily by complete surgical resection, yet 30–55% of patients will develop recurrence and die of the disease. Despite significant advances achieved in the chemotherapy and radiation therapy for advanced disease patients, most patients will eventually develop resistance. Thus, there is a need for novel and effective targeted therapies, the development of which requires our greater understanding of the genetic abnormalities in lung cancers.

As the complexity and unique nature of individual human cancers become apparent, the analysis of individual patients' genetic makeup has become important in guiding the development of novel treatments. A striking example of this has recently emerged in therapies using the epidermal growth factor receptor (EGFR) small molecule inhibitors (Shepherd *et al*, 2005; Tsao *et al*, 2005; Blackhall *et al*, 2006). Somatic mutations in the tyrosine kinase domain and copy number changes of the EGFR gene play critical roles in determining the sensitivity and clinical benefit of NSCLC patients treated by EGFR inhibitor drugs.

The discoidin domain receptors (DDRs) are receptor tyrosine kinases (RTKs) belonging to the same enzyme family as EGFR. The DDR1 gene is divided into 19 exons and has a coding sequence of 2742 bp. Discoidin domain receptor 2 is also divided into 19 exons and encodes a 2568 bp mRNA. Discoidin domain receptor 1 and DDR2 share similar structures, consisting of a characteristic discoidin homology domain, stalk region, transmembrane region, juxtamembrane region, and kinase domain. Compared to other RTKs, DDRs are unique because they have native collagens as their ligands. Discoidin domain receptors have been shown to exhibit altered expression patterns in multiple human cancers, including breast, oesophageal, ovarian, brain and lung tumours (Vogel *et al*, 2006). Of note, DDR1 appears to be preferentially expressed in tumour cells, whereas DDR2 is expressed in tumour stroma (Alves *et al*, 1995). The mechanism by which these receptors may contribute to oncogenesis is as yet unknown; however, given their important role in transmitting signals from the extracellular matrix (ECM), it is possible that they act as regulators of cell proliferation, adhesion, migration, and subsequent tumour metastasis. Similar to EGFR it is conceivable that altered expression and/or mutation of DDRs trigger abnormal activity, ultimately leading to enhanced proliferation and oncogenic transformation. The mutations in EGFR are all found within the catalytic kinase domain, and mostly consist of single-point mutations or small deletions (Sakurada *et al*, 2006; Shigematsu and Gazdar, 2006). In a related study, Davies *et al* (2005) screened for mutations in lung cancer by comprehensively sequencing all 518 kinases in the human genome. Among mutations in several other RTKs, the authors also described novel somatic mutations in DDRs, specifically the mutations A496S and R824W for DDR1 and the mutation R105S for DDR2.

Here, we sought to extensively explore the association of DDRs with human lung cancer by determining their expression levels and the prevalence of the newly reported mutations in a larger cohort of NSCLC primary tumours and tumour cell lines. We demonstrated a significant upregulation of DDR1 and a downregulation of DDR2 in lung tumour tissue compared with matched normal tissues from the same patients; importantly we demonstrated that DDR1 expression was a good prognostic marker for early-stage NSCLC patients. However, we did not identify any of the three previously identified mutations in our cohort of lung cancers or in any of the cell lines tested.

## MATERIALS AND METHODS

### Patients

A total of 146 primary lung tumours and an independent set of 23 matched tumour and normal lung tissue samples were harvested from NSCLC patients treated by surgical resection without adjuvant chemotherapy at the University Health Network and Mount Sinai Hospital, Toronto, Canada (Table 1A). Tissues were harvested within 30 min after complete resection, and the quality and pathology of tumour tissue was confirmed by the study pathologist (M-ST). Samples with a tumour-cell content of at least 50% were used in the study. The University Health Network Research Ethics Board has approved the use of these samples and their associated clinical information in this study.

### Cell lines and xenograft models

Human cell lines (ATCC) were cultured and grown in media according to the manufacturer's recommended conditions (Table 2). A lung xenograft model had been generated previously and no additional animal experiments were necessary for the current work (Liu and Tsao, 1993). Briefly, 2 × 10^6^ cells in 70 *μ*l medium (RPMI-1640, 10% fetal bovine serum) containing 10% Matrigel (BD Bioscience, San Jose, CA, USA) were subcutaneously injected into the ventral abdomen of 5-week-old male severe combined immunodeficiency mice (Table 2). Formation of tumours was observed by palpation, and growth measured twice a week until the tumour reached a volume of 1 cm^3^. After mice were killed, tumour tissue was harvested and snap-frozen in liquid nitrogen. All animals received humane care in compliance with the Guide to the Care and Use of Experimental Animals issued by the Canadian Council of Animal Care. All animal procedures were performed in accordance with a protocol approved by the animal care committee of the University of Toronto.

### RNA extraction

Ribonucleic acid was extracted from patient samples and cell lines using the method of Chomczynski and Sacchi (1987); extracts were purified using the RNeasy kit (Qiagen, Hilden, Netherlands), DNase treated (Ambion, Austin, TX, USA), and quantified via spectrophotometry. Ribonucleic acid quality was assessed by agarose gel electrophoresis.

### Quantification and analysis of DDR expression in lung tumour samples

Quantitative real-time PCR (qRT-PCR) was conducted using the SYBR Green assay in the ABI PRISM 7900-HT (Applied Biosystems, Foster city, CA, USA). Each 10 *μ*l qRT-PCR aliquet contained a 2 ng equivalent of cDNA in a 384-well plate. The reactions were activated at 95°C for 3 min followed by 40 cycles of 95°C (15 s), 65°C (15 s) and 72°C (20 s). The amount of transcript per ng cDNA was calculated using standard curves generated using a pool of genomic DNA from 10 normal lung tissue samples as described (Yun *et al*, 2006). Primer sequences were designed with Primer Express v 2.0 (Applied Biosystems). Primer sequences for DDR1 were forward primer ATGGAGCAACCACAGCTTCTC, reverse primer CTCAGCCGGTCAAACTCAAACT, and for DDR2, forward primer GGAGGTCATGGCATCGAGTT, reverse primer GAGTGCCATCCCGACTGTAATT. Technical replicates displayed high correlation (*R*_avg_=0.95±0.03) and were collapsed by averaging. Expression values were then log_2_-transformed. Standardisation and normalisation were conducted using the geometric mean of the expression levels of four normaliser genes (ACTB, forward primer TCCTAAAAGCCACCCCACTTCT, reverse primer GGGAGAGGACTGGGCCATT, TBP, forward primer GGGCATTATTTGTGCACTGAGA, reverse primer TAGCAGCACGGTATGAGCAACT, BAT1, forward primer CGGTATCAGCAGTTTAAAGATTTTCA, reverse primer TGCCTCGGCCAAATAGGTT, and B2M, forward primer GAGTGCTGTCTCCATGTTTGATGT, reverse primer AAGTTGCCAGCCCTCCTAGAG). An indepth description of this method was published recently (Barsyte-Lovejoy *et al*, 2006).

### PCR and sequencing analysis

Total RNA was transcribed into cDNA using Superscript II Reverse Transcriptase and oligo-dT (Invitrogen, Carlsbad, CA, USA). Primers were designed to amplify the regions containing previously identified DDR mutations in the juxtamembrane (forward primer GAGCTGACGGTTCACCTCTC, reverse primer AATGTCAGCCTCGGCATAAT), and kinase domains (forward primer GGTGCTGATGCTCTGTAGGG, reverse primer CGTGTTGAGTGCATCCTCTG) of DDR1 and the discoidin domain of DDR2 (forward primer GACTTGCACACCCTCCATTT, reverse primer GAGTGGTCGGTGACTGGAAT). The housekeeping gene, glyceraldehyde-3-phosphate dehydrogenase (forward primer CAATGACCCCTTCATTGACC, reverse primer TGCTGTAGCCAAATTCGTTG), was used as a control. cDNA was subjected to PCR amplification consisting of an initial 2 min denaturation at 94°C, followed by 35 cycles of amplification (94°C, 30 s; 57°C, 30 s; 72°C, 45 s), and a final extension at 72°C for 3 min. Polymerase chain reaction products were subjected to agarose gel electrophoresis with appropriate size markers, and samples producing bands of the correct size were purified using the MiniElute PCR Purification kit (Qiagen). Purified samples were sequenced at The Centre for Applied Genomics (TCAG) facility in Toronto, Canada, using an ABI3730XL DNA sequencer. Samples were sequenced in both directions using the same primers as for amplification, and sequences aligned and analysed using ChromasPro software (Technelysium, Tewantin, Australia). Samples that gave ambiguous sequences were repeated up to three times. Two samples were excluded from the study as clean sequences could not be generated.

### Genomic sequencing

Additionally, DNA was extracted from all 12 lung cancer cell lines using standard techniques. Polymerase chain reaction was performed using exon-specific primers spanning putative mutation sites in DDR1 and DDR2, and PCR product sequenced as outlined above.

### Statistical analysis

Matched *t*-tests were used to determine differential expression of DDR1 and DDR2. The Spearman's correlation, Kruskal–Wallis, and Wilcoxon test were used to assess association within and between molecular indices and the pathological or clinical factors. The end points for analyses were overall survival (from date of surgery to date of death) and disease-free survival (from date of surgery to date of disease recurrence). The Cox proportional hazards model was used to test the association of survival and DDR1 expression, where DDR1 expression was treated as a continuous variable. For Kaplan–Meier and multivariate analysis, subjects were dichotomised according to the median expression levels of DDR1 (median=32.6); low DDR1 expressors are defined as patients with expression levels below the median, whereas high DDR1 expressors are those with expression values above the median. Discoidin domain receptor 2 cases were dichotomised based on expressors *vs* nonexpressors. The Cox proportional hazards model was employed for the multivariate analysis. All statistical analysis was conducted using SAS software (v9.1).

## RESULTS

**Figure 1:**
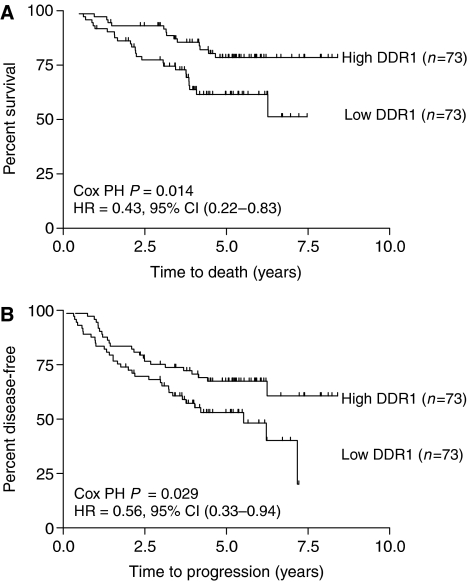
Kaplan–Meier analysis of (**A**) overall survival and (**B**) disease-free survival, according to DDR1 expression levels. Patients were dichotomised based on the median level of DDR1 expression.

Quantitative real-time PCR analysis of DDR1 and DDR2 expression performed on the 146 NSCLC tumour samples (full data set available as Supplementary Table S1) showed neither DDR1 nor DDR2 were associated with factors such as tumour stage (*P*=0.38 and *P*=0.51, respectively), differentiation (*P*=0.93 and *P*=0.60, respectively), and age (Spearman's correlation *P*=0.82 and *P*=0.48, respectively). Normalised DDR1 expression ranged from 0 to 1184 transcript copies per 2 ng of cDNA, whereas DDR2 expression ranged from 0 to 186 copies. The expression of DDR1 was significantly higher in squamous cell carcinoma than in adenocarcinoma (*P*=0.007; median expression 40.6 *vs* 23.1, respectively). In contrast to DDR2 whose expression level was not prognostic (Supplementary Table S2), high expression of DDR1 was associated with significantly favourable overall survival in this cohort of patients (HR=0.43, 95% CI=0.22–0.83, *P*=0.014) (Figure 1A). Discoidin domain receptor 1 expression was also significantly associated with greater disease-free survival (HR=0.56, 95% CI=0.33–0.94, *P*=0.029) (Figure 1B). In multivariate analysis, DDR1 expression remained significant in predicting overall survival (HR=0.5, 95% CI=0.26–0.97, *P*=0.04), but not disease-free survival, after adjustments for stage, differentiation, histology, and age.

**Figure 2:**
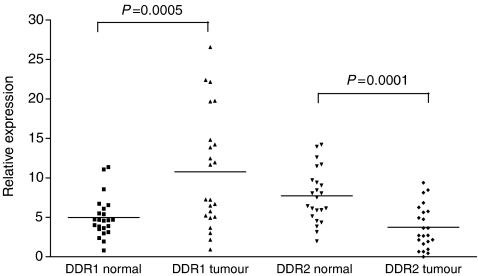
Accumulated plot of DDR qRT-PCR data demonstrating relative expression of DDR1 and DDR2 in normal and cancerous tissues from lung cancer patients. Discoidin domain receptor 1 was found to be significantly upregulated by 2.15-fold (*P*=0.0005) and DDR2 significantly downregulated by an equivalent amount (*P*=0.0001) in tumour tissue compared with normal tissue from lung cancer patients. Expression values were calculated relative to that of four normaliser genes.

**Figure 3:**
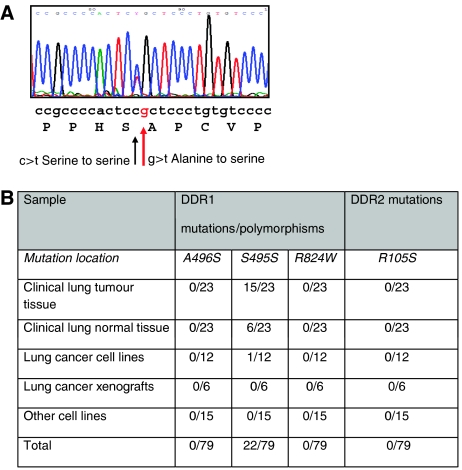
Results of sequence analyses. (**A**) Location of S495S synonymous change adjacent to A496S somatic mutation identified previously. (**B**) Overall prevalence of DDR1 mutations in clinical lung cohort and cell lines. Six patients had the S485S polymorphism in both their normal and tumour tissue.

Next, we analysed a cohort of 23 samples from lung cancer patients, which included matched normal tissue for each tumour sample. Discoidin domain receptor 1 was shown to be upregulated by 2.15-fold (*P*=0.0005), and DDR2 downregulated by an equivalent amount (1.94-fold, *P*=0.0001) in tumour tissue compared with matched normal samples (Table 1B, Figure 2). We then analysed the genetic sequences of DDR1 and DDR2 in this cohort. To our surprise, we failed to detect any of the previously identified mutations in DDR1 and DDR2, nor any new mutations (Davies *et al*, 2005). We then subjected 12 lung cell lines, six lung xenografts, and 15 other cell lines to sequence analysis. The 12 lung cell lines were also subjected to genomic sequencing. Again, none of the previously reported mutations was found. This group included the lung cell line NCI-H1770 that had previously been reported to harbour the R824W mutation in the DDR1 kinase domain (Davies *et al*, 2005) (Figure 3A). We did not detect this mutation in either DNA or RNA sequences from this cell line. Sequences were generated for almost 20% of the coding sequence of DDR1, and 10% of DDR2.

Previously identified polymorphisms located in these regions were successfully identified, confirming the robust nature and specificity of the sequencing method employed. One previously identified polymorphism in DDR1, located at amino acid 495, was identified in 46% of the clinical samples screened. This polymorphism is directly adjacent to the DDR1 A496S mutation identified by Davies *et al* (2005) that was not identified in any sample of our cohort (Figure 3B). This polymorphism is a synonymous change, and is therefore unlikely to be of functional importance; however, it is of interest to note that whereas six of 23 samples harboured this change in both their normal and tumour tissues suggestive of a germline polymorphism, another nine samples recorded the change only in their tumour tissue, indicating an acquired or somatic aetiology.

## DISCUSSION

We have demonstrated for the first time that the expression of DDRs is significantly deregulated in NSCLC. Furthermore we have shown that DDR1 is an independent favourable prognostic marker for early-stage NSCLC patients, and that mutations in DDR1 and DDR2 appear less frequently than previously reported. The collagen-binding RTKs, DDR1 and DDR2, have previously been linked to various human diseases including fibrosis (Alves *et al*, 1995; Mao *et al*, 2002; Lee *et al*, 2004; Avivi-Green *et al*, 2006), atherosclerosis (Hou *et al*, 2001; Hou *et al*, 2002; Ferri *et al*, 2004), and cancer (Johnson *et al*, 1993; Alves *et al*, 1995; Barker *et al*, 1995; Nemoto *et al*, 1997; Weiner *et al*, 2000; Dejmek *et al*, 2003; Ongusaha *et al*, 2003; Heinzelmann-Schwarz *et al*, 2004; Ram *et al*, 2006; Vogel *et al*, 2006). The mechanism by which DDRs may contribute to oncogenesis is as yet unknown.

Although DDR1 has been previously discussed in the context of idiopathic pulmonary fibrosis and inflammation of the lung (Avivi-Green *et al*, 2006; Matsuyama *et al*, 2006a, 2006b), a direct link with lung tumorigenesis has not yet been established. In addition, DDR2 has not yet been investigated in the context of lung cancer. This therefore represents the first study to extensively explore and quantify the expression levels of these unique RTKs in a large clinical cohort of lung cancer patients. Discoidin domain receptor 1 was shown to be upregulated in tumour *vs* normal tissue, whereas DDR2 was downregulated. Intriguingly, our data suggest that higher DDR1 expression can also be a strong independent prognostic indicator for early-stage NSCLC patients. Although DDR2 also appeared to be protective in our cohort, there were ultimately too few cases with quality expression data to make a firm conclusion. These results detailing differences in expression at the mRNA level are also of interest in relation to previous reports that DDR1 exhibits preferential protein expression in tumour epithelial cells, whereas DDR2 is more commonly expressed in the surrounding stroma (Alves *et al*, 1995). At this stage, it remains unknown as to the mechanisms by which DDRs may contribute to oncogenesis.

We were interested to note the recent study reporting for the first time somatic mutations in DDR1 and DDR2 (Davies *et al*, 2005). The authors report mutations in two patient samples (one squamous cell carcinoma and one large cell carcinoma), and one lung cancer cell line (NCI-H1770). However, we could not confirm the existence of the DDR1 mutation in the NCI-H1770 neuroendocrine cell line, which we obtained from ATCC and passaged for three times only. Despite repeated amplification, sequencing and analysis of both forward and reverse sequences, the wild-type DDR1 sequence was observed consistently. Davies *et al* (2005) comment on the unusually large number of mutations in the NCI-H1770 cell line cultured in their laboratory and suggest that this may be indicative of a defect in DNA repair that mimics UV exposure. The apparent discrepancy between the two sets of results may also be owing to differences in clonal strains of the NCI-H1770 cell lines, and passage number at the time of sequence analysis. Furthermore, we amplified and sequenced 33 cell lines (including 12 lung cell lines and six xenografts) and 46 clinical samples (RNA extracted from 23 matched lung tumour and normal tissue) from patients treated at our institution and did not identify any of the three mutations reported by Davies *et al* (2005).

Several explanations exist for this disparity. Lung cancer encompasses a broad range of clinical subtypes, and the makeup of the two cohorts differed. We cannot conclude that differences were owing to our methodology as our PCR and sequencing methods were robust and results repeatable. Further to this point, we managed to detect both germline and acquired polymorphisms in our samples, thereby verifying our ability to detect mutations if they existed in our population. One synonymous nucleotide change in DDR1 was present in almost half of our clinical cohort. This polymorphism is located at amino acid 495, directly adjacent to amino acid 496, previously identified as the site of a somatic mutation in DDR1. The nucleotide change is a cytosine to a thymine change located at the third nucleotide of the codon, resulting in no alteration in the amino acid serine. The previously identified A496S mutation results from a change from a guanine to a thymine (Davies *et al*, 2005). The two altered thymine nucleotides are sequential, suggesting that this region of the DDR1 genome may be a hotspot for genetic variation (Glover *et al*, 2005). We must therefore conclude that if these recently reported DDR1 and DDR2 mutations are valid, then their prevalence must be much lower in the general lung cancer population than expected. This does not however rule out the possibility that they are important mutations contributing clinically to a subset of lung cancers in certain populations. This is analogous to the emerging understanding of the role of EGFR mutations in various populations. As we understand more about the role of RTKs in cancer, it has become important to investigate the presence and prevalence of mutations in these genes, in varied populations. We suggest further research into the presence of mutations in diverse cohorts from varied populations.

This is the first study to show a strong association of the collagen binding RTK, DDR1 with human lung cancer. We have conclusively shown that DDR1 is significantly more (2.15-fold) expressed in tumour as opposed to normal tissues from lung cancer patients, and have demonstrated that it can also be a strong prognostic indicator. Further research is required into the mechanisms by which both DDR1 and DDR2 function in lung carcinogenesis.

## Figures and Tables

**Table 1A:** Comparative clinical and pathological features of the full cohort of NSCLC patients. Patients were dichotomised using the median level of DDR1 expression, whereas patients with undetectable DDR2 were considered low expressors

	**All patients (146)**	**Elevated DDR1 expression (73)**	**Reduced or absent DDR2 expression (104)**
*Histology*			
Adenocarcinoma	92 (63%)	39 (53%)	63 (61%)
Squamous cell carcinoma	54 (37%)	34 (47%)	41 (39%)
			
*Pathological stage*			
Stage I	95 (65%)	51 (70%)	62 (60%)
Stage II	34 (23%)	15 (21%)	30 (29%)
Stage III	17 (12%)	7 (9%)	12 (11%)
			
*T stage*			
T1	45 (31%)	28 (38%)	30 (29%)
T2	92 (63%)	43 (59%)	68 (65%)
T3	9 (6%)	2 (3%)	6 (6%)
			
*N stage*			
N0	94 (64%)	51 (70%)	65 (63%)
N1	38 (26%)	16 (22%)	30 (29%)
N2	14 (10%)	6 (8%)	9 (8%)
			
*Differentiation*			
WD	32 (23%)	18 (25%)	21 (20%)
MD	39 (27%)	21 (29%)	29 (28%)
PD	37 (25%)	16 (22%)	30 (29%)
U	37 (25%)	18 (24%)	24 (23%)
			
*Sex*			
Male	63 (43%)	32 (44%)	47 (45%)
Female	83 (57%)	41 (56%)	57 (55%)
Median Survival time (years)	3.89	4.98	4.29

MD=moderately differentiated; PD=poorly differentiated; WD=well differentiated; U=unknown.

**Table 1B:** Cohort of matched tumour and normal lung tissues with relative DDR1 and DDR2 expression levels. Expression values were calculated relative to that of four normalizer genes

**Tumour**	**Histology**	**Stage**	**T**	**N**	**Differentiation**	**Sex**	**Relative DDR1 expression**	**Relative DDR2 expression**
1	SQ	1B	T2	N0	PD	F	7.25	0.00443
2	SQ	1A	T1	N0	PD	F	5.21	0.0364
3	AD	3A	T2	N2	PD	M	2.16	0.0846
4	AD	2B	T2	N1	PD	F	5.06	0.0626
5	AD	3A	T1	N2	WD	F	22.1	0.0271
6	AD	1A	T1	N0	MD	M	26.5	0.0493
7	AD	1B	T2	N0	WD	M	12.4	0.00648
8	SQ	1B	T2	N0	MD	F	14.2	0.0684
9	AD	3A	T2	N2	PD	F	7.21	0.0563
10	AD	1B	T2	N0	PD	M	3.00	0.0163
11	SQ	1A	T1	N0	PD	M	5.74	0.0264
12	AD	1A	T1	N0	PD	M	0.91	ND
13	SQ	1A	T1	N0	MD	M	22.38	0.0193
14	AD	1B	T2	N0	PD	F	11.66	0.00947
15	SQ	1B	T2	N0	MD	F	6.67	0.0475
16	SQ	1B	T2	N0	MD	F	11.94	0.0575
17	AD	1B	T2	N0	PD	M	6.46	0.0270
18	LCUC	1B	T2	N0	PD	U	3.65	0.0363
19	SQ	1A	T1	N0	MD	M	19.74	0.0213
20	SQ	2B	T2	N1	MD	M	19.66	0.0813
21	LCUC	1B	T2	N0	PD	F	14.82	0.00651
22	AD	1B	T2	N0	PD	F	13.86	0.0215
23	AD	2B	T3	N0	PD	F	4.92	0.0937

AD=adenocarcinoma; DDR1**=**discoidin domain receptor 1; DDR2**=**discoidin domain receptor 2; LCUC=large cell undifferentiated carcinoma; MD=moderately differentiated; ND=expression level below detection limit; PD=poorly differentiated; SQ=squamous cell carcinoma; U=data not available; U=status unknown; WD=well differentiated.

**Table 2:** List of cell lines and xenografts used for sequence analysis

**Name**	**Pathology**
*Lung cell lines*	
A549	AD
H125	ADSQ
H1264	SQ
H157	SQ
H226	SQ
H358	BAC
H460	LCC
H520	SQ
H661	LCC
H1184	SQ
NCI-H1770	NE
RVH6849	AD
	
*Lung xenografts*	
MGH4	LCC
MGH7	SQ
MGH8	AD
MGH13	AD
MGH24	AD
MGH30	ADSQ
	
*Other cell lines*	
NTERA2	Testicular cancer
HTB126	Breast
HepG2	Hepatoblastoma
HT1080	Fibrosarcoma
SW872	Liposarcoma
T47D	Breast carcinoma
MCF7	Breast carcinoma
MCF12A	Breast
Colo205	Colon carcinoma
MOLT-4	Leukaemia
SKOV3	Ovarian adenocarcinoma
SKMEL28	Melanoma
MCF10A	Breast
HEK293	Kidney
MDAMB231	Breast carcinoma

AD=adenocarcinoma; ADSQ=adenosquamous carcinoma; BAC=bronchoalveolar carcinoma; LCC=large cell carcinoma; SQ=squamous cell carcinoma; NE=neuroendocrine tumour.
