# Supplementary material for: Expression and mutation analysis of the discoidin domain receptors 1 and 2 in non-small cell lung carcinoma
Source: Br J Cancer. 2007 Feb 13;96(5):808–14. doi: 10.1038/sj.bjc.6603614 (PMC2360060; doi:10.1038/sj.bjc.6603614)
Supplement: Supplementary Table S2 [file 6603614x2.doc]

**Supplementary Table, Table S2**:

Univariate and multivariate significance for the expression of DDR1 and DDR2 in overall and disease-free survival.

|  | **Univariate Analysis** | | | | **Multivariate Analysis** | | | |
| --- | --- | --- | --- | --- | --- | --- | --- | --- |
|  | **Overall Survival** | | **Disease-Free Survival** | | **Overall Survival** | | **Disease-Free Survival** | |
|  | p-value | HR (95% CI) | p-value | HR (95% CI) | p-value | HR (95% CI) | p-value | HR (95% CI) |
| DDR1 | 0.014 | 0.43 (0.22-0.83) | 0.029 | 0.56 (0.33-0.94) | 0.04 | 0.5 (0.26-0.97) | 0.06 | 0.57 (0.31-1.03) |
| DDR2 | 0.16 | 0.57 (0.26-1.24) | 0.19 | 0.67 (0.37-1.22) | 0.45 | 0.69 (0.26-1.82) | 0.63 | 0.84 (0.41-1.72) |

HR, Hazard Ratio
